# Analysis of COVID-19 data using neutrosophic Kruskal Wallis H test

**DOI:** 10.1186/s12874-021-01410-x

**Published:** 2021-10-17

**Authors:** Rehan Ahmad Khan Sherwani, Huma Shakeel, Wajiha Batool Awan, Maham Faheem, Muhammad Aslam

**Affiliations:** 1 College of Statistical and Actuarial Sciences, University of the Punjab Lahore, Lahore, Pakistan; 2 Department of Statistics, Faculty of Science, King Abdulaziz University, Jeddah, 21551 Saudi Arabia

**Keywords:** Neutrosophic, Fuzzy logic, Kruskal Wallis H test, Covid-19

## Abstract

**Background:**

Kruskal-Wallis H test from the bank of classical statistics tests is a well-known nonparametric alternative to a one-way analysis of variance. The test is extensively used in decision-making problems where one has to compare the equality of several means when the observations are in exact form. The test is helpless when the data is in an interval form and has some indeterminacy.

**Methods:**

The interval-valued data often contain uncertainty and imprecision and often arise from situations that contain vagueness and ambiguity. In this research, a modified form of the Kruskal-Wallis H test has been proposed for indeterminacy data. A comprehensive theoretical methodology with an application and implementation of the test has been proposed in the research.

**Results:**

The proposed test is applied on a Covid-19 data set for application purposes. The study results suggested that the proposed modified Kruskal-Wallis H test is more suitable in interval-valued data situations. The application of this new neutrosophic Kruskal-Wallis test on the Covid-19 data set showed that the proposed test provides more relevant and adequate results. The data representing the daily ICU occupancy by the Covid-19 patients were recorded for both determinate and indeterminate parts. The existing nonparametric Kruskal-Wallis H test under Classical Statistics would have given misleading results. The proposed test showed that at a 1% level of significance, there is a statistically significant difference among the average daily ICU occupancy by corona-positive patients of different age groups.

**Conclusions:**

The findings of the results suggested that our proposed modified form of the Kruskal-Wallis is appropriate in place of the classical form of the test in the presence of the neutrosophic environment.

## Introduction

Hypothesis testing is a scientific process used to investigate the acceptance or rejection of a proposition under consideration. Two approaches are used in statistics to verify a hypothesis: 1) Parametric approach 2) Nonparametric approach. The most important aspect of the parametric approach is the satisfaction of the assumption about data’s normality, and a few tests require the equality of population variances [1]. In most situations, the distributional assumption under a parametric test hardly satisfy, and the use of nonparametric or distribution-free tests is a common practice [2, 3]. However, all such nonparametric tests apply to data containing determined observations. In real life, there are various scenarios where we have non-precise data, and in such cases, the existing hypothesis testing approach based on classical test statistics cannot be implemented. Recent studies have suggested nonparametric tests based on interval-valued data and fuzzy logic [4]. Smarandache [5] generalized the fuzzy logic in the neutrosophic sense by considering the interval-valued data and the measure of indeterminacy or falseness. Smarandache introduced Neutrosophic Statistics as a generalization of classical statistics applied when the data under consideration is in neutrosophic numbers [6]. Smarandache and Khalid [6] verified the efficiency of neutrosophic logic. Several authors have implemented neutrosophic logic for data containing uncertainty and vagueness; see ref [7–11].

Furthermore, several authors have developed statistical tests to analyze fuzzy data; see, for example, refs [12–15]. Also, in fuzzy logic and neutrosophic statistics, several research works have been contributed by introducing decision-making analysis for the data set containing uncertainty and vagueness [16–18]. Recently, Aslam introduced different statistical tests using Neutrosophic Statistics, including the tests of homogeneity of variance for uncertainty environment, the goodness of fit test in the presence of uncertain parameters, and the Kolmogorov-Smirnov tests under uncertainty [19–21].

In 1952, Kruskal and Wallis [12] provided a robust rank-based test for the *k* sample problem as an alternative to the parametric approaches, such as the one-way analysis of variance (ANOVA). Kruskal-Wallis H test has been used for analysis purposes in various manners; for example, see refs [13–18]. In the classical k sample problem, data are determined and do not contain any ambiguity or vagueness. However, in many current scientific studies, the observations are not necessarily relentless, and indeterminate parts quantitatively express the uncertainties in a sample. The existing Kruskal-Wallis H test cannot be used to investigate the data which is measured in the neutrosophic intervals. A detailed literature review has given a shred of clear evidence that no such test is available that can be useful as a nonparametric alternate for ANOVA under an indeterminate environment. The unavailability of a method for the said purpose is a source of motivation for the current research. The goal is to develop a test that compares several sample observations or group(s); the proposed test is easy to apply and understandable. The proposed modified Kruskal-Wallis test results in the interval-valued form and is preferable for data containing vagueness and uncertainty. The objectives of this article are (1) to introduce the modified neutrosophic Kruskal Wallis test; (2) to define the methodology of the neutrosophic Kruskal Wallis test; and (3) to compare the performance of the existing Kruskal Wallis test with the proposed test through an application on Covid-19 data set under Neutrosophy. More information about the application of neutrosophic statistics can be seen in [22–24].

The article is planned as follows. Section 2 presents the computational method for the application of the neutrosophic Kruskal Wallis test. In section 3, the modified Kruskal Wallis test has been demonstrated with an eloquent example of the Covid-19 data set for scrutinizing its efficiency and competence. It is anticipated that the modified nonparametric Kruskal Wallis test will proficiently analyze the data in the presence of uncertainty and vagueness as compared to the existing Kruskal Wallis test under classical statistics. Finally, the results are discussed and generalized with some conclusive remarks.

## Computational method of the modified Kruskal Wallis test under uncertainty

In Classical Statistics, nonparametric tests are methods of statistical analysis that do not require a distribution to meet the assumptions necessary to be analyzed. These tests apply to non-normal data sets. Due to this reason, they are sometimes referred to as distribution-free tests. The basic purpose of suggesting the Kruskal Wallis test is to scrutinize that all independent samples containing neutrosophic observations come from neutrosophic populations with equal means implying that the populations under uncertainty are identical. The proposed nonparametric test is applicable for data where the measure of uncertainty or the measure of falseness has been recorded. Suppose *X*_*N*_ = *a*_*N*_ + *b*_*N*_*I*_*N*_; *X*_*N*_ ∈ [*X*_*L*_, *X*_*U*_] is a neutrosophic number where the first part represents the measure of determinacy and the second part represents the measure of vagueness or uncertainty. For *I*_*N*_ ∈ [*I*_*L*_, *I*_*U*_] = 0, the neutrosophic number reduces to a random variable under classical statistics. The neutrosophic variable *X*_*N*_ represents the neutrosophic sample obtained from the population containing imprecise, uncertain, and indeterminate observations; for detail, see ref [5].

### Modified Kruskal Wallis H test

Under Classical Statistics, the Kruskal-Wallis H test is used to test the null hypothesis that all k independent samples come from populations having equal means against the alternative hypothesis that at least one population varies. The existing nonparametric test is a generalization of the two-sample Mann-Whitney U test. It is an extremely useful test when the assumptions of normality do not hold, or the population variances are not equal, but helpless for data under uncertainty. The modified Kruskal Wallis test under uncertainty will be applicable under the following assumptions:

1. The data consists of uncertain, imprecise, and indeterminate values.
2. The neutrosophic samples must be random.
3. The two neutrosophic samples must be mutually independent.
4. The test is generally considered robust to ties, but if there are ties in the data set, they shouldn’t be concentrated together in one part of the sample.

Suppose we have *k*_*N*_ independent neutrosophic samples of sizes *n*_1*N*_, *n*_2*N*_, …, *n*_*kN*_ (∑*n*_*iN*_ = *n*_*N*_). Let *X*_*iN*_ (*X*_*i*1*N*_, *X*_*i*2*N*_, *X*_*i*3*N*_, …, *X*_*inN*_) represents the neutrosophic observations of the *ith* sample. To perform this test under uncertainty, arrange all the *n*_*N*_ observations containing uncertainty of the *k*_*N*_ samples combined in ascending order of magnitude and assign the ranks to them. In the case of ties, assign the average of the ranks. To distinguish the neutrosophic sample observations, let the letters *A*_*N*_, *B*_*N*_, *C*_*N*, …_ represent the sample observations of the first, second, and third neutrosophic samples, respectively. The observations of the neutrosophic samples are replaced with their corresponding ranks. Add these ranks for each sample and denote the sums by *R*_1*N*_, *R*_2*N*_, …, *R*_*nk*_. Now compute

1 $${S}_{kN}^2=\sum_{i=1}^{k_N}\frac{R_{iN}^2}{n_{iN}};{R}_N\in \left[{R}_L,{R}_U\right];{n}_N\in \left[{n}_L,{n}_U\right];{k}_N\in \left[{k}_L,{k}_U\right]$$

and

2 $${S}_{rN}^2=\sum_{i,j}{r}_{ijN}^2;$$

where *r*_*ijN*_ is the rank assigned to neutrosophic observation *X*_*ijN*_; *X*_*ijN*_ ∈ [*X*_*ijL*_, *X*_*ijU*_].If there are no ties, then

3 $${S}_{rN}^2=\frac{n_N\left({n}_N+1\right)\left(2{n}_N+1\right)}{6}$$

The modified Kruskal-Wallis statistic *H*_*N*_; *H*_*N*_ ∈ [*H*_*L*_, *H*_*U*_] is given by

4 $${H}_N=\frac{\left({n}_N-1\right)\left({S}_{kN}^2-{C}_N\right)}{\left({S}_{rN}^2-{C}_N\right)}$$

where *C*_*N*_; *C*_*N*_ ∈ [*C*_*L*_, *C*_*U*_] denotes the appropriate correction term given by

5 $${C}_N=\frac{n_N{\left({n}_N+1\right)}^2}{4}$$

In case of no ties, the neutrosophic statistic *H*_*N*_ becomes

6 $${H}_N=\frac{12{S}_{kN}^2}{n_N\left({n}_N+1\right)}-3\left({n}_N+1\right)$$

The neutrosophi form of the proposed test *H*_*N*_ ∈ [*H*_*L*_, *H*_*U*_] can be expressed as follows

7 $${H}_N={H}_L+{H}_U{I}_{NH};{I}_{NH}\in \left[{I}_{LH},{I}_{UH}\right]$$

Note here that the proposed statistic *H*_*N*_ ∈ [*H*_*L*_, *H*_*U*_] is a generalization of the existing test under classical statistics. The first part *H*_*L*_ shows the determined part, *H*_*U*_*I*_*NH*_ denoted the indeterminate part and *I*_*NH*_ ∈ [*I*_*LH*_, *I*_*UH*_] is the measure of indeterminancy/uncertainty. The proposed test reduces to the existing test when *I*_*LH*_ =0.

The neutrosophic Kruskal Wallis *H*_*N*_ test is used to test the null hypothesis that all *k*_*N*_ populations have identical distributions. For a large value of the test statistic under uncertainty is rejected. For example, only three samples have five or fewer neutrosophic observations; the significance of this test statistic is determined by using Kruskal and Wallis’ Table [19] having critical values for all combinations of sample sizes up to 5,5,5. In case one of the neutrosophic samples contains more than five observations, or there are more than five observations in each neutrosophic sample and the null hypothesis is true, the neutrosophic test statistic *H*_*N*_ follows a chi-square distribution with (k-1) degrees of freedom.

## Application of the proposed modified Kruskal Wallis H test

**Fig. 1:**
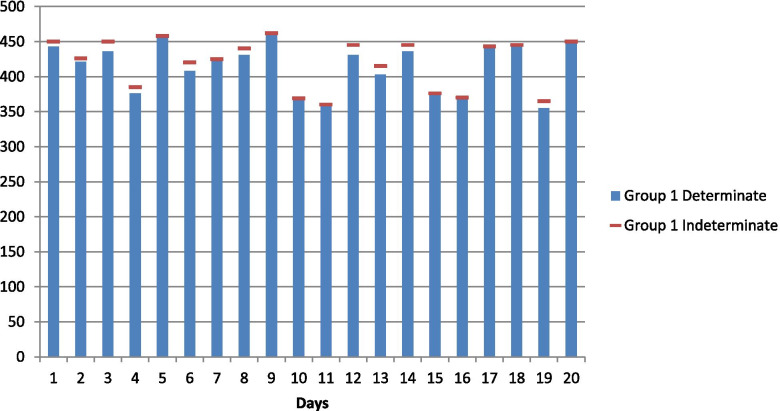
Daily ICU occupancy of Covid-19 patients aged 55 and above

**Fig. 2:**
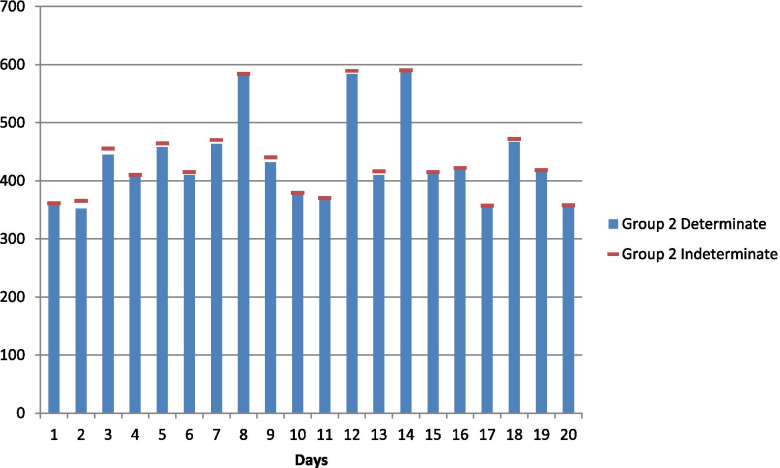
Daily ICU occupancy of Covid-19 patients aged 35–55

**Fig. 3:**
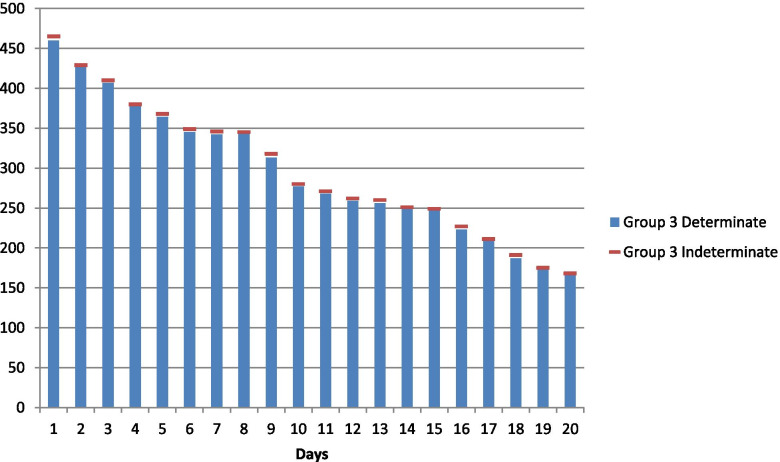
Daily ICU occupancy of Covid-19 patients aged 35 and below

For applying the proposed neutrosophic Kruskal Wallis test, data representing the daily ICU occupancy by Corona-positive patients have been considered, which was recorded specifically from Pakistan. The hypothesis under investigation for the research is to test a statistically significant the difference in the daily ICU occupancy of Covid-19 patients based on their age groups. Neutrosophy or uncertainty is introduced in the data for a better illustration. The neutrosophic Kruskal Wallis test is applied to test the null hypothesis that there is no difference in the daily ICU occupancy of Covid-19 patients among different age groups in Pakistan during December 2020. Daily ICU occupancy of Covid-19 patients aged 55 and above are shown in Fig. 1, Daily ICU occupancy of Covid-19 patients aged 35–55 are shown in Fig. 2 and Daily ICU occupancy of Covid-19 patients aged 35 and below are shown in Fig. 3.

The neutrosophic null and alternate hypotheses for neutrosophic data given in Table 1 are: The average daily ICU occupancy of Covid-19 patients for all three age groups are equal against the alternative hypothesis that the average daily ICU occupancy of Covid-19 patients for at least two of the three age groups are not equal. Table 1 contains data on daily ICU occupancy of Corona-positive patients by three different age groups. By combining and arranging the data in ascending order and assigning ranks to them, it is found that ties exist in the neutrosophic data set for both determinate and indeterminate parts. Therefore, the neutrosophic statistic is given in (4) applies to this data set containing the measure of uncertainty. Here *R*_1*N*_ = [757.5, 766.5], *R*_2*N*_ = [775.5, 767], *R*_3*N*_ = [297, 296.5], where *R*_1*N*_, *R*_2*N*_ *and R*_3*N*_ represents the sum of ranks of age groups 1, 2, and 3, respectively.

**Table 1:** Daily ICU occupancy of Covid-19 patients in Pakistan in December 2020

Day/Age	55 and above	35–55	35 and below
1	[443, 450]	[359, 361]	[460, 465]
2	[421, 426]	[352, 365]	[427, 429]
3	[436, 450]	[445, 455]	[407, 410]
4	[376, 385]	410	[378, 380]
5	[458, 458]	[458, 464]	[364, 368]
6	[408, 420]	[410, 415]	[345, 349]
7	[422, 425]	[463, 470]	[342, 346]
8	[431, 440]	[580, 584]	345
9	[459, 462]	[432, 440]	[313, 318]
10	369	379	[277, 280]
11	360	370	[268, 271]
12	[431, 445]	[584, 589]	[259, 262]
13	[403, 415]	[410, 416]	[256, 260]
14	[436, 445]	[587, 590]	251
15	376	415	249
16	370	[419, 422]	[223, 227]
17	443	357	[209, 211]
18	445	[467, 472]	[187, 191]
19	[355, 365]	[415, 418]	[173, 175]
20	450	358	168

Source: Our World in Data COVID-19

From (1) and (2), we have

$${S}_{kN}^2=\sum_{i=1}^{k_N}\frac{R_{iN}^2}{n_{iN}}=\left[63170.78,63186.18\right]$$

and

$${S}_{rN}^2=\sum_{i,j}{r}_{ijN}^2=\left[73803.5,73802\right]$$

From (4)

$${H}_N=\frac{\left({n}_N-1\right)\left({S}_{kN}^2-{C}_N\right)}{\left({S}_{rN}^2-{C}_N\right)}=\left[24.12,24.17\right]$$

$${p}_N- value=\left[0.000011,0.000011\right]$$

Assuming the level of significance to be 1%, the critical region is *H*_*N*_ > *χ*^2^_0.01,2_ = 9.21. Since the calculated value of test statistic based on neutrosophic observations lies in the critical region (*p*-value < α), we, therefore, reject the neutrosophic null hypothesis and conclude that daily ICU occupancy of different age groups of Covid-19 patients is not equal.

## Advantages of the proposed test

In this section, the efficiency of the proposed test *H*_*N*_ ∈ [*H*_*L*_, *H*_*U*_] will be compared with the existing test under classical statistics in terms of a measure of uncertainty. As mentioned earlier, the neutrosophic *H*_*N*_ = *H*_*L*_ + *H*_*U*_*I*_*NH*_; *I*_*NH*_ ∈ [*I*_*LH*_, *I*_*UH*_] has consisted of determinate (the existing test) and indeterminate parts. The neutrosophic form of *H*_*N*_ ∈ [*H*_*L*_, *H*_*U*_] for the real data is expressed as: *H*_*N*_ = 24.12 + 24.17*I*_*NH*_; *I*_*NH*_ ∈ [0,0.002]; where the first value 24.12 shows the results of the existing test when *I*_*LH*_ =0 and 24.17*I*_*NH*_ is an indeterminate part. Note that the measure of indeterminacy associated with the test *H*_*N*_ ∈ [*H*_*L*_, *H*_*U*_] is 0.002. From the study, it can be seen that the proposed test the result of the test statistic in the range of 24.12 to 24.17. On the other hand, the existing test provides only the determined/exact value of the test. In addition, the proposed test *H*_*N*_ ∈ [*H*_*L*_, *H*_*U*_] gives information about the measure of uncertainty. Based on the information, the proposed test can be interpreted as follows: when the level of significance *α* =0.05, the chance of rejecting the null hypothesis when it is true is 0.05, the probability of accepting the null hypothesis is 0.95 with the chance of uncertainty of 0.002. From the comparisons, it can be concluded that the proposed test *H*_*N*_ ∈ [*H*_*L*_, *H*_*U*_] gives more information about the test. In addition, the proposed test is flexible, adequate, and effective to be applied in uncertainty as compared to the existing test.

## Conclusion and discussion

This article proposed the modified form of the rank-based nonparametric Kruskal Wallis H test for observations containing the measure of uncertainty or the measurement of falseness when comparing *k* samples. It is evident from Table 1 that uncertain data used for the illustration purpose reduces to the determined part under classical statistics if no observations of uncertainty are logged. For example, for sample one, the first observation 443 for the first group represents the determinate part of the indeterminate interval. The second value, which is 450, represents the indeterminate part of the interval. We can observe here that the modified Kruskal-Wallis test results in the indeterminacy interval rather than the determined values, and this implies that the proposed test provides a good measure of uncertainty. Recent studies also show that the methods dealing with the interval-valued data are more suitable in the indeterminate environment than classical statistical techniques [25, 26]. The work was originally motivated by the extensive research work under the fuzzy logic and neutrosophic statistics used for the interval-valued data set. The proposed nonparametric test can be readily applied to compare k samples testing the hypothesis that they have equal means.

The application of this new neutrosophic Kruskal-Wallis test on the Covid-19 data set showed that the proposed test provides more relevant and adequate results. The data representing the daily ICU occupancy by the Covid-19 patients were recorded for both determinate and indeterminate parts. The existing nonparametric Kruskal Wallis H test under Classical Statistics would have given misleading results. The proposed test showed that at a 1% level of significance, there is a statistically significant difference among the average daily ICU occupancy by corona-positive patients of different age groups.

The modified Kruskal Wallis test can be used to compare the averages of several sample observations or group(s); the proposed test is easy to apply and understandable. The Neutrosophic Kruskal Wallis test results in an uncertain interval, which is ideal when the data is measured from the complex system. The application of the proposed test is recommended for different fields, including biomedical sciences, engineering, and many other statistical areas. On the other hand, applying nonparametric tests under classical statistics on the data containing vagueness can produce misleading results. In conclusion, the proposed neutrosophic nonparametric test provides an efficient tool to data analysts for analyzing k samples in the presence of uncertainty and indeterminacy. However, more properties of this modified Kruskal-Wallis test can be derived for future research. The evaluation of the proposed test using different measures can be studied as future research.

## Data Availability

All data generated or analysed during this study are included in this published article.

## References

[1] Higgins JJ (2004). An introduction to modern nonparametric statistics.

[2] Krzywinski M, Altman N (2014). Nonparametric tests. Nat Methods.

[3] Chan Y (2003). Biostatistics 102: quantitative data–parametric & nonparametric tests. Blood Press.

[4] Buckley JJ (2005). Fuzzy statistics: hypothesis testing. Soft Comput.

[5] Smarandache F (2010). Neutrosophic Logic-A Generalization of the Intuitionistic Fuzzy Logic. Multispace & Multistructure Neutrosophic Transdisciplinarity (100 Collected Papers of Science).

[6] Smarandache, F., Khalid, H. E. & Essa, A. K. Neutrosophic Logic: the Revolutionary Logic in Science and Philosophy. (Infinite Study, 2018).

[7] Nabeeh NA, Abdel-Basset M, El-Ghareeb HA, Aboelfetouh A (2019). Neutrosophic multi-criteria decision making approach for iot-based enterprises. IEEE Access.

[8] Abdel-Basset M, Nabeeh NA, El-Ghareeb HA, Aboelfetouh A (2020). Utilising neutrosophic theory to solve transition difficulties of IoT-based enterprises. Enterprise Inform Syst.

[9] Abdel-Baset M, Chang V, Gamal A (2019). Evaluation of the green supply chain management practices: a novel neutrosophic approach. Comput Ind.

[10] Abdel-Basset M, Atef A, Smarandache F (2019). A hybrid Neutrosophic multiple criteria group decision making approach for project selection. Cogn Syst Res.

[11] Broumi, S., Bakali, A., Talea, M. & Smarandache, F. Bipolar neutrosophic minimum spanning tree. (Infinite Study, 2018).

[12] Kruskal WH, Wallis WA (1952). Use of ranks in one-criterion variance analysis. J Am Stat Assoc.

[13] McKight, P. E. & Najab, J. Kruskal-wallis test. The corsini encyclopedia of psychology, 1–1 (2010).

[14] Hecke TV (2012). Power study of anova versus Kruskal-Wallis test. J Stat Manage Syst.

[15] MacFarland, T. W. & Yates, J. M. in Introduction to nonparametric statistics for the biological sciences using R 177–211 (Springer, 2016).

[16] Soltani N, Safajou F, Amouzeshi Z, Zameni E (2017). The relationship between body image and mental health of students in Birjand in 2016 academic year: a short report. J Rafsanjan Univ Med Sci.

[17] Lou, Y., Yuen, S. Y. & Chen, G. in Proceedings of the Genetic and Evolutionary Computation Conference Companion. 1337–1341.

[18] Muremi, L. & Bokoro, P. in 2018 IEEE International Conference on Environment and Electrical Engineering and 2018 IEEE Industrial and Commercial Power Systems Europe (EEEIC/I&CPS Europe). 1–4 (IEEE).

[19] Aslam M (2021). A new goodness of fit test in the presence of uncertain parameters. Complex Intell Syst.

[20] Aslam M (2019). Introducing Kolmogorov–Smirnov tests under uncertainty: an application to radioactive data. ACS Omega.

[21] Aslam M (2020). Design of the Bartlett and Hartley tests for homogeneity of variances under indeterminacy environment. J Taibah Univ Sci.

[22] Chen J, Ye J, Du S (2017). Scale effect and anisotropy analyzed for neutrosophic numbers of rock joint roughness coefficient based on neutrosophic statistics. Symmetry.

[23] Aslam M (2019). Neutrosophic analysis of variance: application to university students. Complex Intelligent Syst.

[24] Smarandache F (2014). Introduction to neutrosophic statistics: infinite study.

[25] Meyer JP, Seaman MA (2013). A comparison of the exact Kruskal-Wallis distribution to asymptotic approximations for all sample sizes up to 105. J Exp Educ.

[26] Chen J, Ye J, Du S, Yong R (2017). Expressions of rock joint roughness coefficient using neutrosophic interval statistical numbers. Symmetry.

